# Therapeutic targeting of the TPX2/TTK network in colorectal cancer

**DOI:** 10.1186/s12964-023-01290-2

**Published:** 2023-09-28

**Authors:** Hibah Shaath, Radhakrishnan Vishnubalaji, Ramesh Elango, Dinesh Velayutham, Puthen Veettil Jithesh, Nehad M. Alajez

**Affiliations:** 1 Translational Cancer and Immunity Center (TCIC), Qatar Biomedical Research Institute (QBRI), Hamad Bin Khalifa University (HBKU), Qatar Foundation (QF), PO Box 34110, 00000 Doha, Qatar; 2 College of Health & Life Sciences, Hamad Bin Khalifa University (HBKU), Qatar Foundation (QF), Doha, Qatar

**Keywords:** Colorectal cancer, Precision medicine, TPX2, TTK, DDX39A, LRP8, Gene-drug-interaction

## Abstract

**Background:**

While the increased screening, changes in lifestyle, and recent advances in treatment regimen have decreased colorectal cancer (CRC) mortality, metastatic disease and recurrence remains a major clinical challenge. In the era of precision medicine, the identification of actionable novel therapeutic targets could ultimately offer an alternative treatment strategy for CRC.

**Methods:**

RNA-Seq was conducted using the illumina platform, while bioinformatics analyses were conducted using CLC genomics workbench and iDEP.951. Colony forming unit, flow cytometry, and fluorescent microscopy were used to assess cell proliferation, cell cycle distribution, and cell death, respectively. The growth potential of CRC cells under 3-dimensional (3D) conditions was assessed using Matrigel. STRING database (v11.5) and Ingenuity Pathway Analysis (IPA) tool were used for network and pathway analyses. CRISPR-Cas9 perturbational effects database was used to identify potential therapeutic targets for CRC, through integration with gene-drug interaction database. Structural modeling and molecular docking were used to assess the interaction between candidate drugs and their targets.

**Results:**

In the current study, we investigated the therapeutic potential of targeting TPX2, TTK, DDX39A, and LRP8, commonly upregulated genes in CRC identified through differential expression analysis in CRC and adjacent non-cancerous tissue. Targeted depletion of TPX2 and TTK impaired CRC proliferation, cell cycle progression, and organoid formation under 3D culture conditions, while suppression of DDX39A and LRP8 had modest effects on CRC colony formation. Differential expression analysis and bioinformatics on TPX2 and TTK-deficient cells identified cell cycle regulation as the hallmark associated with loss of TPX2 and TTK. Elevated expression of TPX2 and TTK correlated with an oncogenic state in tumor tissue from patients with colon adenocarcinoma, thus corroborating an oncogenic role for the TPX2/TTK network in the pathogenesis of CRC. Gene set enrichment and pathway analysis of TPX2^high^/TTK^high^ CRC identified numerous additional gene targets as integral components of the TPX2/TTK network. Integration of TPX2/TTK enriched network with CRISPR-Cas9 functional screen data identified numerous novel dependencies for CRC. Additionally, gene-drug interaction analysis identified several druggable gene targets enriched in the TPX2/TTK network, including AURKA, TOP2A, CDK1, BIRC5, and many others.

**Conclusions:**

Our data has implicated an essential role for TPX2 and TTK in CRC pathogenesis and identified numerous potential therapeutic targets and their drug interactions, suggesting their potential clinical use as a novel therapeutic strategy for patients with CRC.

Video Abstract

**Supplementary Information:**

The online version contains supplementary material available at 10.1186/s12964-023-01290-2.

## Background

Despite the many advances in colorectal cancer (CRC) management, it remains among the most common malignancies in both men and women worldwide, contributing to the overall increase in cancer disease burden and mortality. It is estimated that 50% to 60% of CRC patients will succumb to metastatic disease, while the 5-year survival for those patients is approximately 14% [1]. In the era of precision medicine, recent research efforts aim to provide a better understanding of the molecular alterations in CRC and to provide patients with novel targeted therapeutic interventions, as single agents or in combination with standard therapies, such as chemotherapy, radiotherapy and surgery. As our understanding of the complexity of cancer networks expands, so do the potential routes used for targeted therapy. The current landscape for CRC-targeted therapy includes targeting the epidermal growth factor receptor (EGFR) and its related pathways using monoclonal antibodies, such as cetuximab and panitumumab. However, early studies have shown no significant improvement in overall survival (OS) in patients with *RAS* mutations, this is in addition to CRC molecular heterogeneity [2], highlighting the importance of tailored therapy. More promising outcomes were observed when cetuximab was used in combination with other therapies, including BRAF (such as vemurafenib or dabrafenib) and HER2 inhibitors (trastuzumab) [3]. Our recent investigations have unraveled the transcriptional portraits of CRC from the Middle East and North Africa region and have identified numerous deregulated networks in CRC [4–7].

In the current study, we aimed to delve deeper and identify potential therapeutic targets based on our recent transcriptomic profiling of CRC*.* Our data identified Targeting protein for Xklp2 (TPX2) and TTK Protein Kinase (TTK), among others, as potential therapeutic targets for CRC, highlighting an essential role for those two genes in the regulation of cell cycle processes and as essential genes for CRC proliferation and organotypic growth. Concordantly, a previous study by We et al., reported TPX2 as novel biomarker for CRC growth and metastasis [8], while Takahashi et al., reported the AURKA/TPX2 axis to play an oncogenic role in colon cells through cooperation with MYC [9]. Zhang et al., also reported TTK to predict unfavorable prognosis and to regulated proliferation of CRC cells [10]. In the current study, expression of TPX2 and TTK was associated with oncogenic state in colon adenocarcinoma (COAD), while employing gene set enrichment analysis we constructed the gene-drug network for potential therapeutic targeting of TPX2/TTK networks and their potential implementation in CRC targeted therapy.

## Methods

### Transcriptomic profiling of CRC cohort

Transcriptomic profiling methodology and the clinical characteristics of our local CRC cohort are described in details in our previous publication [6].

### Maintenance of cancer cell lines

Human CRC (HCT116 and HT-29 cell lines) were cultured in Dulbecco’s modified Eagle’s medium (DMEM). All culture media were supplemented with 10% fetal bovine serum (FBS) and 1% penicillin/streptomycin (Thermo Scientific Inc., Rockford, IL, USA). Cells were cultured as an adherent monolayer at 37°C under 5% CO2 in a humidified incubator.

### siRNA transfection of CRC cells

To investigate the functional role of selected gene targets in CRC, HCT116 and HT-29 cell lines were transfected with the indicated SMARTpool siRNAs and non-targeting control purchased from Dharmcon (Lafayette, CO, USA). Transfection was performed using a reverse transfection protocol, where siRNAs at a final concentration of 30 nM were diluted in 50 µL of Opti-MEM (s11058-021; Gibco, Carlsbad, CA, USA), and 1.5 µL of Lipofectamine 2000 (cat. no. 52758; Invitrogen) were diluted in 50 µL Opti-MEM. The diluted siRNAs and Lipofectamine 2000 were mixed together and incubated at ambient temperature for 20 min. Depending on the plate format, 800 µl of transfection mixture were added to 6-well tissue culture plate, followed by the addition of 2.4 ml of HCT116 or HT-29 (0.168 × 10^6^ cells/mL) in transfection medium. Alternatively, 200 µl of transfection mixture were added to each well in a 12-well tissue culture plate, and subsequently 600 µl of HCT116 or HT-29 (0.168 × 10^6^ cells/mL) in transfection medium were added to each well. Twenty-four hours later, each well was topped up with transfection medium (complete DMEM without antibiotics).

### Total RNA library preparation and NGS

The total RNA library preparation and sequencing procedure was followed as described before [11]. Total RNA samples from transfected cells with a RIN > 8 were used as input for the library preparation using TruSeq Stranded Total RNA Library Prep Gold kit (Cat #: 20020598) from Illumina following the manufacturer’s protocol. In brief, 100 ng of total RNA was subjected to rRNA depletion and then to fragmentation. The first-strand cDNA synthesis was achieved with random hexamers and SuperScript II Reverse Transcriptase (Cat#: 18064014) from ThermoFisher Scientific. The second cDNA strand synthesis was performed by substituting dTTP with dUTP. The double-stranded cDNA is then end-repaired and adenylated. Barcoded DNA adapters were ligated to both ends of the double-stranded cDNA was then amplified. The quality of constructed libraries was assessed on an Agilent 2100 Bioanalyzer system and were quantified using Qubit 2.0 fluorometer (Invitrogen). The libraries were pooled, clustered on a cBot platform, and sequenced on an Illumina HiSeq 4000 at a minimum of 50 million paired end reads (2 × 75 bp) per sample.

### Bioinformatics and network analyses

FASTQ files were subsequently mapped and aligned to the hg38 reference genome using built in RNA-seq analysis module in CLC genomics workbench 20.0 with default settings. Normalized transcript per million (TPM) expression values were subsequently subjected to differential expression analysis using 2.0-fold change and < 0.05 false discovery rate (FDR)-adjusted *p*-value cut-off in Altanalyze v.2.1.3. Transcripts with raw expression values < 1.0 TPM were excluded from the analysis. Hierarchical clustering was performed using cosine for columns and cosine for rows. For network analysis, genes of interest were used as input into the STRING v 11.5 database to highlight known protein–protein interactions (PPI) between the corresponding proteins based on text mining, experiments, databases, co-expression, neighborhood, genes fusion, and co-occurrence evidence. Differentially expressed genes in (DEGs) TPX2^high^ vs TPX2^low^ and TTK^high^ vs TTK^low^ were imported into the ingenuity pathway analysis (IPA) software (Ingenuity Systems, Qiagen) and were used for functional annotations and network analysis using canonical, upstream regulator, and downstream effector analyses. The *p*-value is the negative log of P and represents the possibility of focus genes in the network being found together by chance. IPA analyses were conducted as described before [12, 13].

### Colony Formation Unit (CFU) assay

The proliferative ability of HCT116 and HT-29 cells under the indicated treatment conditions was determined using a clonogenic assay using Crystal Violet, (ACS reagent, ≥ 90.0% anhydrous basis, Sigma). Cells were seeded in 12 well plate and on day 5 post siRNA-mediated depletion of TPX2, TTK, DDX39A, and LRP8, wells were washed and stained. Once dry, the plates were imaged, and the number of colonies was observed under an inverted microscope. To quantify the data, the wells were covered with 10% SDS solution and left on a rocker to dissolve the crystal violet. Once completely dissolved, 200 µl of the solution was transferred to a 96 well plate (in duplicates) and absorbance was taken using a plate reader (NanoQuant, Infinite M200 Pro, Tecan).

### Cell cycle analysis using flow cytometry

Cell cycle analyses were conducted on HCT116 and HT-29 cells post siRNA transfection. On day 4 post transfection, any floating cells were collected and pooled with the adherent trypsinized cells, followed by washing with PBS and fixing with 70% ethanol, added dropwise while cells were on ice. Cells were then stored at -20°C overnight. Before staining, cells were washed with PBS twice and incubated in RNase A (100 mg/mL) and propidium iodide (PI; 50 mg/mL) staining solution and then subjected to cell cycle analysis using BD LSRFortessa X-20 flow cytometer (BD Biosciences, CA, USA) using the FL3 channel. FCS files were exported and analyzed using FlowJo software (FlowJo 10.7.2 © Becton Dickinson & Company (BD)).

### Detection of cell death using fluorescence microscopy

The acridine orange/ethidium bromide (AO/EtBr) fluorescence staining method was used to assess apoptosis/necrosis in treated CRC cells vs. control. In brief, CRC cells in 12-well flat-bottom tissue culture plate were washed twice with PBS on day 4 post transfection, and were subsequently stained with dual fluorescent staining solution containing 100 mg/mL AO and 100 mg/mL EtBr (AO/EtBr, Sigma Aldrich, St. Louis, MO, USA) for 2 min; subsequently, the cells were observed and imaged under an Olympus IX73 fluorescence microscope (Olympus, Tokyo, Japan). The differential uptake of AO/EtBr allows the identification of viable and non-viable cells. Principally, AO/EtBr was used to visualize the number of cells that had undergone apoptosis, while EtBr-positive cells (red) indicated necrotic cells.

### 3D organoid culture

Cells were harvested on day 4 post transfection with the indicated siRNAs and were suspended in Corning Matrigel® Matrix®. Using 6 cm dishes, matrigel containing cells were pipetted into the center of the dish forming 3 separate cell containing domes. Dishes were placed in a humidified incubator to facilitate polymerization of the Matrigel® (37 °C, 20 min) to solidify the domes and growth medium (DMEM) was then added to the dish, covering the domes. Organoid formation and growth were monitored periodically and images were taken on day 7 using EVOS cell imaging system.

### Retrieval of RNA-Seq data and bioinformatics analysis

Raw RNA sequencing data were retrieved from the sequence read archive database under accession no. PRJNA413956, consisting of 10 CRC and 10 normal tissues (NT). The Kallisto index was constructed by creating de Bruijn graph employing the GENCODE release (V33) reference transcriptome and 31 length k-mer. FASTQ files were subsequently pseudo aligned to the generated index using KALLISTO 0.4.2.1, as previously described [7, 14]. Differential expression analyses were conducted using AltAnalyze v.2.1.3 software as we described before [15].

The gene expression data (TPM) in COAD from the cancer genome atlas (TCGA) dataset were retrieved from the UCSC https://xena.ucsc.edu/. Differential expression and GO enrichment analysis were conducted using iDEP.951 (http://bioinformatics.sdstate.edu/idep95/). Briefly, the normalized TPM expression values were imported into iDEP.951 and were subjected to log transformation, and low abundant transcripts (< 1 TPM) were excluded from the analysis. The cohort was then divided into high and low based on TPX2 or TTK median expression. DEGS (1.5 fold-change (fc) and *p* < 0.05 FDR-adjusted) were then subjected to GO enrichment analysis in iDEP.951, as described before [16]. Oncoprint for the eight genes investigated in the current study was generated using the cBioPortal database as described before [17]. Stage-plot and survival analysis were conducted using GEPIA2 database as described before [5, 18].

### Construction of gene-drug interaction networks

The identified gene list from TPX2/TTK network from COAD was imported into the drug gene interaction database (https://www.dgidb.org/). The gene-drug interaction matrix was then imported into Cytoscape v3.10 and networks were constructed as described before [19, 20]. Briefly, the drug IDs were used as sources and gene IDs were used as targets. The edges indicated the type of interaction. Only drugs with inhibitory effects were used to construct the network.

### Structural modeling and molecular docking

In this study we employed molecular docking to investigate the binding interactions of TTK kinase with three drugs: BAY-1161909 (Bay-11), BAY-1217389 (Bay-12), and Hesperadin (HES). The protein–ligand docking study involved the preparation of the structures using Openbabel, a widely used software for molecular structure conversion and preparation [21]. Subsequently, Site finder tools were used to find the potential active residues of selected protein, and then electrostatic surface maps were created around these residues to define docking sites [22]. The MOE tools were used to perform the docking of proteins with three compounds and subsequently a triangular algorithm was used to find the 10 best poses of docking molecules [23]. We applied London dg function and force fields refinement algorithms to create 10 best poses per molecule and then minimized them. These top 3 molecules were selected based on their binding energies for further interaction analysis with active residues of selected protein. LigX tool of MOE was used to prepare the two-dimensional (2D) plots of receptor-ligand interactions focused on electrostatic/non-electrostatic interactions, hydrogen bonding, and hydrophobic interactions. For complexes, these interactions assist in validating the binding pattern of the compounds within the active site of the protein [23].

To analyze and visualize the docking results, the widely employed molecular visualization tool Pymol was used (https://pymol.org/2/#page-top). Pymol enabled the interactive and 3D representation of the protein–ligand complexes, facilitating the examination of ligand binding orientations and interactions within the protein's active site. The sources of 3D structures are given in Table S1.

### Statistical analyses

Statistical analyses and graphing were performed using Microsoft Excel 365 and GraphPad Prism 9.0 software (GraphPad, San Diego, CA, USA) or as an integral part of the computational pipelines. The Benjamini–Hochberg FDR method within AltAnalyze was used to calculate adjusted *p* values.

## Results

### Identification and functional validation of selected essential genes in CRC

**Fig. 1:**
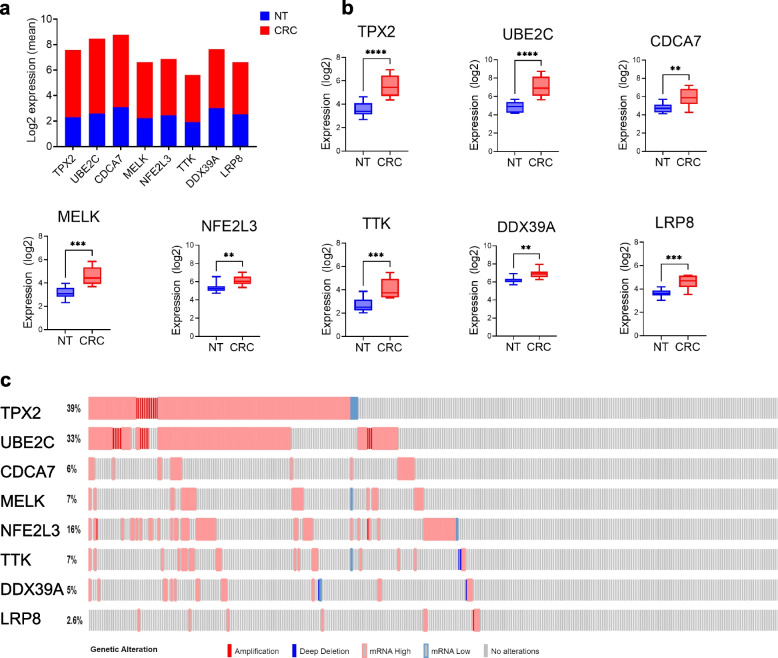
Expression of selected genes in CRC. **a** Elevated expression of TPX2, UBE2C, CDCA7, MELK, NFE2L3, TTK, DDX39A, and LRP8 in CRC compared to normal tissue (NT) from our local cohort. **b** Validation of the expression of the same gene panel in CRC compared NT from the PRJNA413956 CRC dataset (**c**) Oncoprint illustrating the expression of the eight selected genes in COAD from the TCGA dataset. Color legend indicated gene alterations

Our initial analysis identified eight potential therapeutic targets based on their upregulated expression in CRC from our previous studies [4, 6] and the limited studies on their potential role in CRC. The expression of the eight selected genes (TPX2, UBE2C, CDCA7, MELK, NFE2L3, TTK, DDX39A, and LRP8) in CRC and adjacent non-tumor tissue (NT) is illustrated in Fig. 1a. Concordantly, the expression of the same gene panel was validated in the PRJNA413956 dataset (Fig. 1b) as well as in COAD TCGA dataset using cBioPortal as shown in Fig. 1c, which confirmed their elevated expression in COAD, thus corroborating their potential use as therapeutic targets. The correlation between the expression of the indicated genes and clinical staging and survival is provided in Figs. S1, S2, and S3.

### Targeted depletion of TPX2, TTK, DDX39A, and LRP8 reduces CRC cell proliferation

**Fig. 2:**
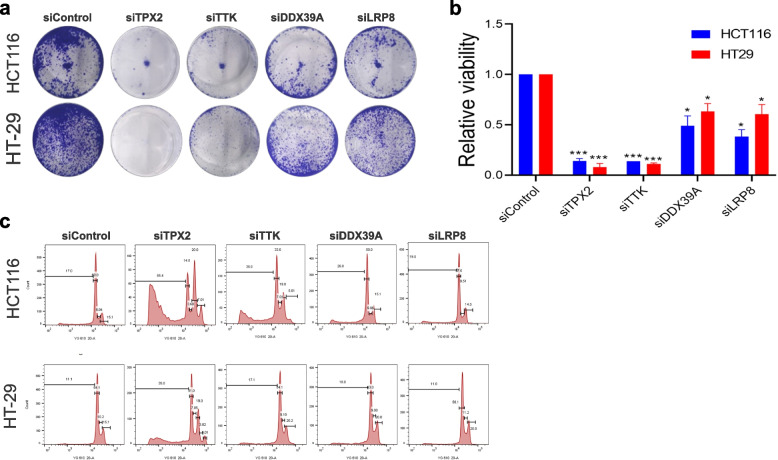
Targeted depletion of TPX2, TTK, DDX39A and LRP8 impairs CRC proliferation. **a** Representative CFU images of HCT116 and HT-29 cells on day 7 post-knockdown of the indicated genes. **b** Quantitative analysis of CFU formation in HCT116 and HT-29 from (**a**) under different treatment conditions. Data are presented as mean ± S.E.M., *n* = 4.. *p < *0.05*, ***p < 0.0005. **c** Representative cell cycle analysis of HCT116 and HT-29 under the indicated treatment conditions

To gain a better understanding of the potential role of the identified gene targets in CRC, the expression of the respective genes was suppressed in HCT116 and HT-29 CRC models employing siRNA-mediated gene silencing, and subsequently, we assessed their ramifications on CRC cellular proliferative. Suppression of TPX2 and TTK impaired CRC CFU potential in both HCT116 and HT-29 CRC models, which was less remarkable upon DDX39A and LRP8 suppression (Fig. 2a). Quantitative analysis of CFU data under different treatment conditions is provided in Fig. 2b. Suppression of UBE2C, CDCA7, MELK, and NFE2L3 had modest effects on CRC proliferation (Fig. S4).

We subsequently employed FACS analysis to assess the effects of TPX2, TTK, DDX39A, and LRP8 depletion on the cell cycle progression of CRC cells. Our data revealed the most remarkable effects for TPX2 and TTK depletion, where the cells exhibited impairment in the progression through the cell cycle compared to the control siRNA-transfected cells (Fig. 2c). Suppression of DDX39A and LRP8 also affected cell cycle distribution, although not as remarkable as TPX2 and TTK suppression (Fig. 2c). A higher number of cells were detected in the sub G0/G1 (apoptotic) and polyploidy in HCT116 and HT-29 CRC cells depleted of TPX2 and TTK (Fig. 2c). The percentages of cells in each stage of the cell cycle under each treatment condition are provided in Table S2.

### Effects of TPX2, TTK, DDX39A, and LRP8 depletion on CRC cell viability

**Fig. 3:**
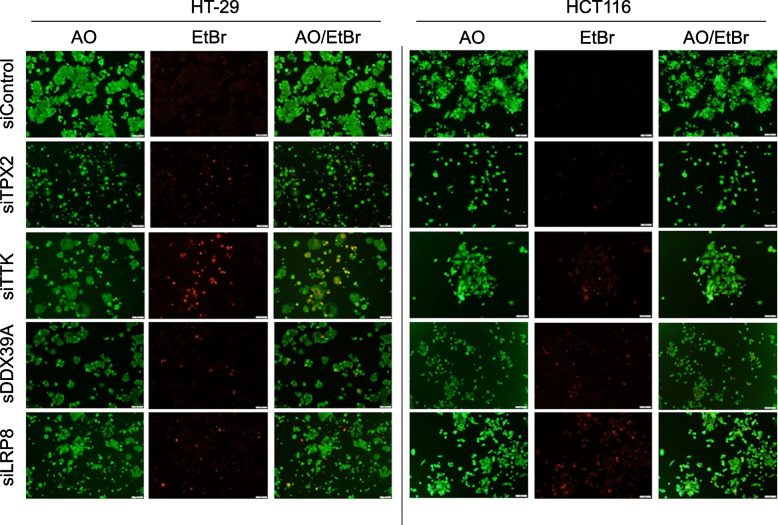
Dead-live staining of HT-29 and HCT116 CRC models in response to TPX2, TTK, DDX39A and LRP8 depletion. Representative fluorescence images for CRC cells post-siRNA-mediated knockdown of TPX2, TTK, DDX39A or LRP8. Cells were stained on day 4 with AO/EtBr to detect live (green) cells (first panel) and dead cells (red; necrotic) shown in the second panel. Third panel shows the merged images

To explore the mode of cell death and morphological changes in response to siRNA-mediated gene silencing of TPX2, TTK, DDX39A and LRP8, we subsequently employed the dead-live AO/EtBr staining assay as we described before [13]. Concordant with the cell cycle data, the cellular growth was reduced in both TPX2 and TTK-depleted CRC cells, employing the HCT116 and HT-29 CRC models (Fig. 3). AO staining of treated cells revealed chromatin shrinkage compared to siRNA control treated cells. Increased numbers of necrotic cells, staining positive with EtBr (red), were also seen in TPX2 and TTK depleted CRC cells (Fig. 3). Notably, we observed a fewer number of cells to stain with EtBr in the siTPX2 condition, possibly due to its devastating effects on survival and proliferation, in addition to the fact that many of these cells had lifted off the plate at the time of analysis. DDX39A and LRP8 depletion also increase the number of dead cells, concordant with CFU data.

### Suppression of TPX2 and TTK impairs CRC growth under 3D organotypic culture condition

**Fig. 4:**
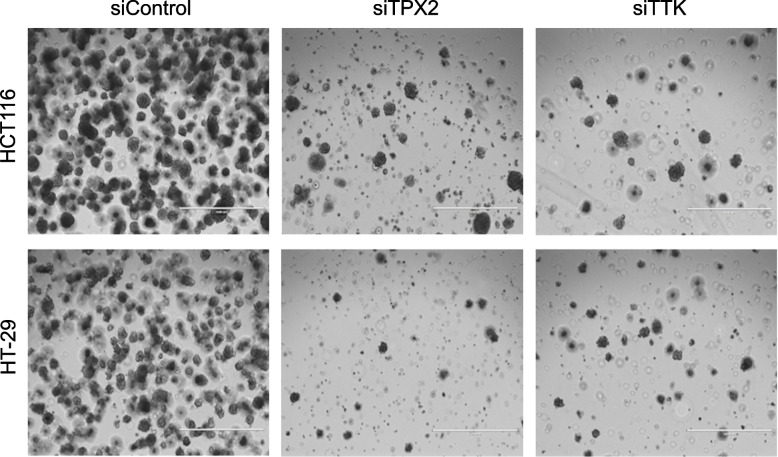
Effects of TPX2 and TTK depletion on 3D organoid formation of CRC cells. Representative images of organoid formation by CRC cell models post-siRNA-mediated knockdown of TPX2 and TTK, compared to siRNA control treated cells. Cells were imaged on day 7 post transfection

Given their remarkable effects on CRC proliferation and cell cycle progression, the remaining experiments were focused on TPX2 and TTK. To assess the effects of TPX2 and TTK depletion on the ability of CRC cells to form colonies under conditions that recapitulate the 3D tumor environment, HCT116 and HT-29 were treated with siRNAs targeting TPX2, TTK, or control siRNAs and were subsequently grown in matrigel under 3D culture conditions. Data presented in Fig. 4 revealed significant suppression of organoid formation of both cell models in response to TPX2 and TTK depletion, thus highlighting an essential role for the two genes for CRC survival under 2D and 3D culture conditions. In addition to the reduced number of colonies, organoids formed by TPX2 and TTK depleted CRC cells were considerably smaller in size and showed less cell integrity when compared to organoids formed by siRNA control treated cells.

### Transcriptional landscape of TPX2 and TTK depleted CRC cells

**Fig. 5:**
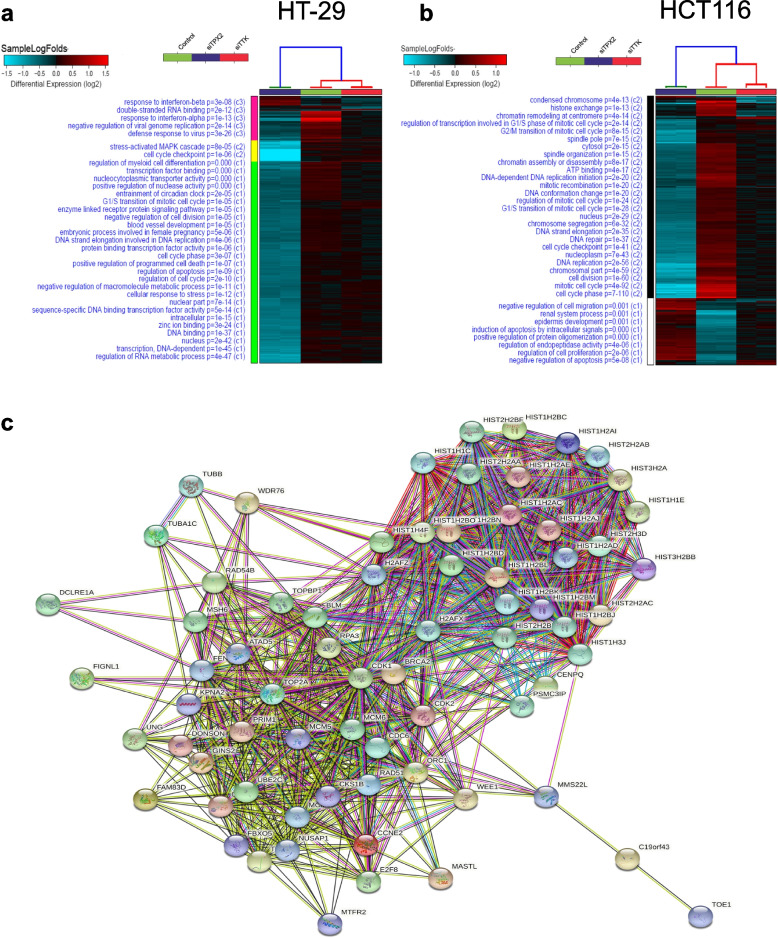
Transcriptional alterations and affected gene ontology (GO) functional categories in TPX2 and TTK depleted CRC models. Transcriptional alterations in TPX2 and TTK depleted cells in HT-29 (**a**) and HCT116 (**b**) CRC cell models. The text on the left indicates the enriched functional categories (GO). **c** STRING PPI network analysis on the commonly downregulated genes in both HT-29 and HCT116 cells depleted of TPX2

To gain more insight into the role of TPX2 and TTK in promoting CRC tumorigenesis, HCT116 and HT-29 CRC cells were transfected with siRNAs targeting the respective genes and were subsequently subjected to whole transcriptome analysis on day 3 post transfection. HT-29 cells depleted of TPX2 and TTK showed profound changes in gene expression compared to the control cells (Fig. 5a, and Tables S3 and S4). Gene ontology (GO) enrichment analysis revealed regulation of cell cycle (cell cycle checkpoint, G1/S transition, DNA strand elongation), and regulation of apoptosis, as the most suppressed GO functional categories in TPX2 and TTK depleted HT-29 cells (Fig. 5a). This data would be concordant with presented data from the CFU, cell cycle, 3D organoid culture, and AO/EtBr staining.

Similarly, RNA-Seq analysis of HCT116 model also highlighted a drastic effect on global transcription patterns after TPX2 and TTK depletion (Fig. 5b and Tables S5 and S6). Remarkably, the downregulation of TPX2 suppressed several biological functions, including cell division, DNA replication DNA repair, G1/S transition, DNA strand elongation, and ATP binding. TPX2 and TTK depleted cells shared similar downregulation of transcripts related to histone exchange, chromatin condensation and remodeling, and shared downregulated negative regulation of cell migration (Fig. 5b). Figure 5c illustrates the PPI network among common downregulated genes in TPX2 depleted cells for both cell models using STRING database, mostly affecting cell cycle processes.

### Differential expression and gene set enrichment analysis in TPX2^high^ and TTK^high^ COAD

**Fig. 6:**
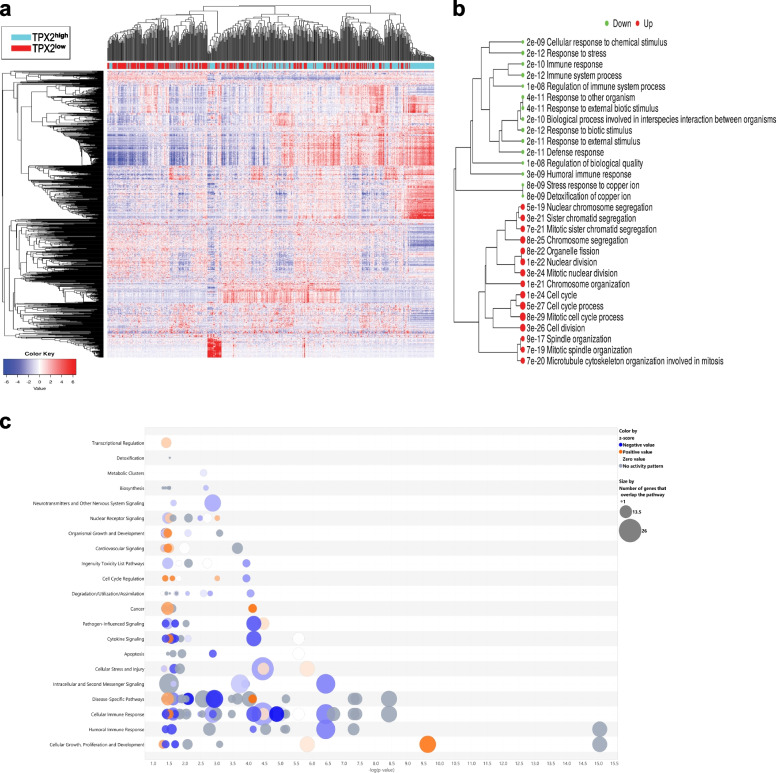
Differential expression and gene set enrichment analysis in TPX2^high^ COAD. **a** Heat map clustering of 512 COAD transcripts stratified into high vs low according to median TPX2 expression. **b** GO enrichment tree in the TPX2^high^ vs TPX2^low^ COAD. Enrichment *p* value is indicated for each functional category. **c** Bubble chart illustrating activated (orange) and suppressed (blue) canonical pathways in TPX2^high^ vs TPX2^low^ COAD employing IPA analysis. Size of the bubble corresponds to the number of genes that overlap the pathway. X-axis represents the -log *p*-value

To gain a better understanding of the function of TPX2 and TTK in the context of CRC tumors, we retrieved transcriptomic data from the TCGA COAD cohort (*n* = 512) and grouped the patients into high vs. low based on median TPX2 or TTK expression. Differential expression analysis (1.5 fc, < 0.05 FDR) revealed distinct clustering of TPX2^high^ vs TPX2^low^ COAD, thus corroborating functional differences in relation to TPX2 expression (Fig. 6a). GO enrichment analysis revealed an association between elevated expression of TPX2 and functional categories related to mitotic cell cycle, while COAD with reduced TPX2 expression were more associated with immune responses as illustrated in the GO enrichment tree (Fig. 6b). Concordantly, IPA analysis on the DEGS in the TPX2^high vs^ TPX2^low^ group revealed strong enrichment in canonical pathways related to cellular growth, proliferation, and development as illustrated in Fig. 6c. Highest enrichment was for genes in SPINK1 General Cancer and the Kinetochore Metaphase Signaling Pathway, which would be concordant with the known role for TPX2 in mitosis (Table S7).

**Fig. 7:**
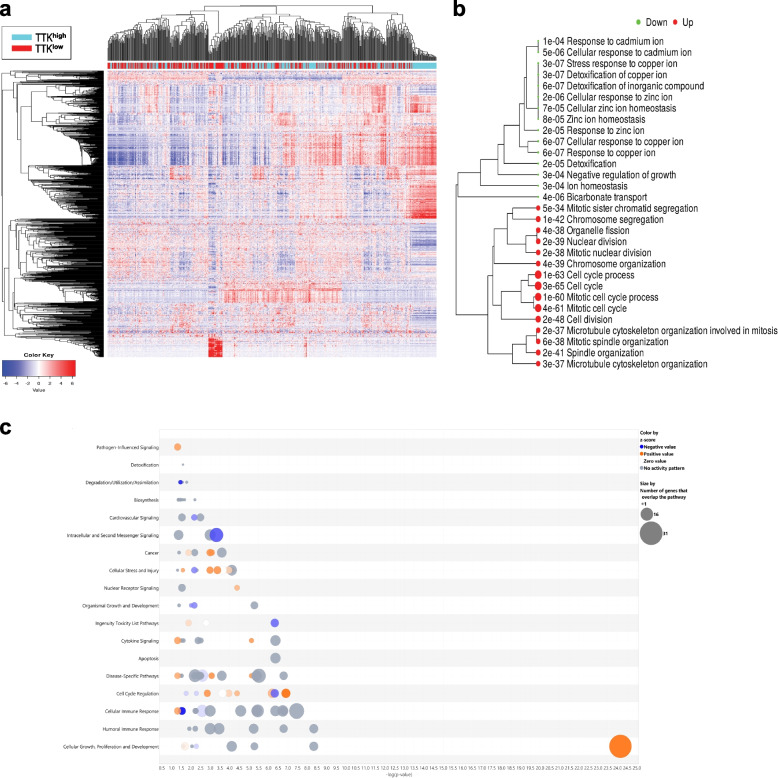
Differential expression and gene set enrichment analysis in TTK^high^ COAD. **a** Heat map clustering of 512 COAD transcripts stratified according to median TTK expression. **b** GO enrichment tree in the same cohort of COAD according to median TTK expression. Enrichment *p* value is indicated for each functional category. **c** Bubble chart illustrating activated (orange) and suppressed (blue) canonical pathways in TTK^high^ vs TTK^low^ COAD employing IPA analysis. Size of the bubble corresponds to the number of genes that overlap the pathway. X-axis represents the -log *p*-value

Similar patterns of clustering were also seen in TTK^high^ vs. TTK^low^ COAD (Fig. 7a). GO enrichment tree highlighted activation of cell cycle associated processes as the hallmark of TTK^high^ COAD (Fig. 7b). IPA analysis on the DEGS in TTK^high^ vs TTK^low^ COAD revealed significant association with cellular growth, proliferation and development canonical pathway as illustrated in Fig. 7c. Highest enrichment was for genes belonging to the Cell Cycle Control of Chromosomal Replication and Kinetochore Metaphase Signaling canonical pathways (Table S8). Taken together, our data highlighted significant enrichment in cell cycle processes as the hallmark of TPX2^high^ and TTK^high^ COAD.

### Construction of TPX2/TTK enriched networks and their drug interactions

**Fig. 8:**
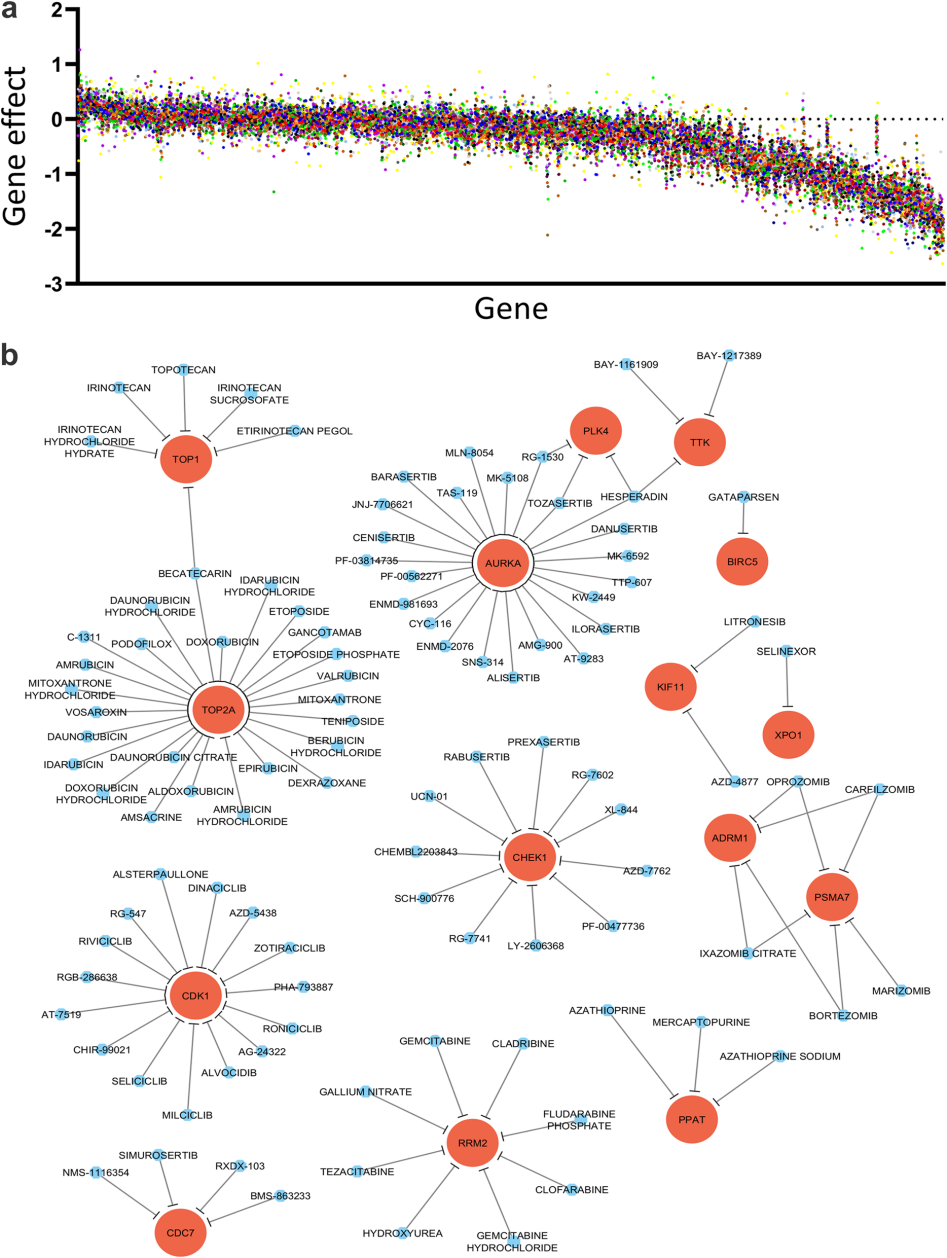
CRC dependency map and their drug interactions in TPX2/TTK enriched networks. **a** Dot plot illustrating the essentiality of 498 identified genes from TPX2/TTK enriched networks, employing CRISPR-Cas9 functional screening data. X-axis represent the gene, y-axis resent the gene effect score, while each dot represents a single CRC cell model. **b** Network illustrating the interaction between the indicated genes and corresponding drugs retrieved from the drug-gene interaction database

We subsequently sought to identify the TPX2 and TTK enriched network in CRC and explore their therapeutic potential, given the limited number of small molecule inhibitors targeting both genes. Data presented in Figs. 6 and 7 had identified 498 genes associated with TPX2 or TTK expression in COAD (Table S9). To explore the therapeutic potential of TPX2/TTK enriched networks, we retrieved essentiality gene data from CRISPR-Cas9 functional screening in CRC cell models from the Achilles project [24]. Data presented in Fig. 8a depicts the gene effect score for each of the identified gene targets in a panel of CRC cell models. We subsequently explored the Drug Gene Interaction Database and identified currently available small molecule inhibitors targeting genes from the TPX2/TTK network (Table S10). Our analysis identified three currently available drugs targeting TTK, however no drugs were found to target TPX2 (Fig. 8b). Nonetheless, our analysis identified numerous drugs targeting other components of the TPX2/TTK network, including small molecules targeting AURKA, TOP2A, CDK1, ADRM1, TOP1, PSMA7, RRM2, KIF11, CHEK1, CDC7, XPO1, PPAT, PLK4, and BIRC5 (Fig. 8b), suggesting targeting those genes as an alternative therapeutic strategy for CRC.

### Structural modeling and molecular docking of TTK interacting drugs

**Fig. 9:**
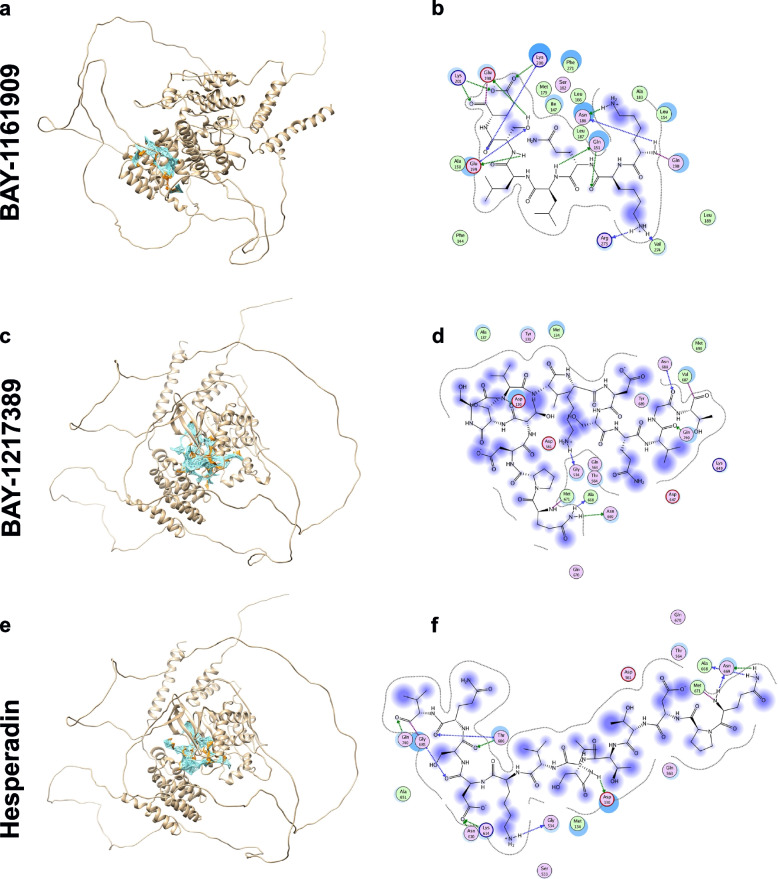
Structural modeling and molecular docking of TTK interacting drugs. Docking pose of TTK protein in gold with the corresponding drugs in cyan (**a**) Bay-11, **c** Bay-12 and **e** HES. 2D plots of receptor-ligand interactions are shown with their amino acid positions represented as beads for (**b**) Bay-11, **d** Bay-12 and **f** HES. Hydrophobic amino acids (Ala, Met, Val, Leu, Ile, Phe) in green, other amino acids in pink while charged amino acids (Asp, Glu, Lys, Arg) are presented with dark rings

To provide more in-depth understanding of the interaction between TTK and the three identified drugs (BAY-1161909 (Bay-11); BAY-1217389 (Bay-12); and Hesperadin (HES)), molecular docking simulations were employed to predict the binding affinity and modes of ligands within the active site of TTK. Our results reveal unique binding poses for each drug and shed light on their potential interactions with TTK kinase (Fig. 9a-f). Zoomed images of the interactions between the three drugs and TTK are shown in Fig. S5. All three molecules demonstrated a strong binding interaction with TTK kinase with Bay-11, Bay-12, and HES exhibiting an affinity of -12.08, -11.84, and -13.49 kcal/mol respectively. Based on our investigation, HES exhibited the highest binding affinity and most favorable fit, suggesting stable interactions within TTK active site.

## Discussion

Given the aggressive nature and limited therapeutic options for CRC, in this study, we explored the therapeutic potential of several candidate genes identified through our differential expression analysis of CRC and adjacent unaffected tissue [4, 6, 7]. Our current data identified TPX2, TTK, DDX39A, and LRP8 as essential genes in CRC, where targeted depletion of those genes impaired cell proliferation, 3D organoid formation, and cell cycle progression. Transcriptomic profiling of TPX2 and TTK depleted CRC cells corroborated a role for both genes in cell cycle regulation, which was further validated in a large cohort of COAD transcriptomic data from the TCGA dataset.

TPX2, encoded by the *TPX2* gene, is one of the spindle assembly factors and plays a key role in microtubule assembly during the M phase of the cell cycle. TPX2 recruits a plus-end directed motor protein, Xlp2, a protein that is required in early mitosis and localizes to spindle poles, to microtubule minus ends of asters. Because of its integral role in microtubule assembly and therefore mitosis, TPX2 is found to be overexpressed in different types of human cancers including hepatocellular carcinoma [25], medullary thyroid cancer [26], bladder carcinoma [27, 28], prostate cancer [29] and estrogen receptor-positive metastatic breast cancer [30], contributing to tumor growth and metastasis. In one study, TPX2 was significantly overexpressed in 129 of 203 (60.8%) CRC metastatic lesions when compared to normal tissue. TPX2 overexpression also correlated with clinical staging, vessel invasion, metastasis, and worse overall and metastasis free survival [8]. Such differential expression of TPX2 offers a therapeutic window in CRC. Consistent with our findings, TPX2 suppression inhibited proliferation and tumorigenicity, migration and invasion ability of CRC cells both in vitro and in vivo*;* mechanistically associated with AKT-mediated MMP2 activity [8]. Our recent data also implicated a role for TPX2 in triple negative breast cancer (TNBC) [31]. Concordantly, Matson et al., reported high TPX2 nuclear expression to strongly correlate with high-grade morphology, elevated clinical stage, negative ER and PR status, and with both disease-specific and overall survival. Increased TPX2 nuclear expression was also correlated with elevated ploidy, supernumerary centrosomes, and TP53 mutation [32].

The elevated expression of TPX2 was also reported in multiple cancer types. For instance, high TPX2 expression correlated with poor prognosis in prostate cancer. Depletion of TPX2 significantly inhibited cell viability and migration in vitro as well as tumor growth in vivo. Interestingly, the observed phenotypic changes were restored after rescuing TPX2, which could be further inhibited using the CDK1 inhibitor, RO-3306 [33]. Employing multiple datasets, elevated expression of TPX2 was implicated in multiple cancer types, including Hepatic cell cancer [34], lung adenocarcinoma [35], and non-small cell lung cancer [36], thus corroborating an essential function for TPX2 in multiple cancers.

The second investigated gene in our study, TTK, encodes a dual protein kinase that can phosphorylate tyrosine, serine and threonine residues, first identified through screening T cell expression libraries with anti-phosphotyrosine antibodies. TTK is a highly conserved kinase with the highest expression in highly proliferative cells such as the testis and thymus [37]. Survival and tumor recurrence data related to TTK expression levels in gastric cancer patients found TTK expression to negatively correlate with survival and tumor recurrence, while the knockdown of TTK inhibited proliferation and increased apoptosis. Mechanistically, TTK was found to regulate the proliferation and apoptosis of tumor cells through the Akt-mTOR pathway, where TTK knockdown inhibited its activation [38]. Due to its essential role in chromosome alignment at the centromere during mitosis and its requirement for centrosome duplication, high TTK expression was observed in multiple cancer types, including neuroblastoma, where TTK was associated with poor overall survival. Silencing TTK promoted cell apoptosis via the caspase-dependent mitochondrial apoptotic pathway, where cells underwent mitotic catastrophe following polyploidization/aneuploidization [39]. Interestingly, we observed similar findings in CRC, where TTK depleted cells were largely apoptotic and showed an increased percentage of polyploidy. Other studies also reported elevated expression of TTK in prostate cancer [40] and lung adenocarcinomas [41].

Our gene-drug interaction analysis identified three drugs targeting TTK, namely: BAY-1161909, BAY-1217389, and Hesperadin. Modeling and docking analyses revealed strong interactions between the three identified drugs and TTK. Interestingly, BAY-1161909, BAY-1217389 were previously shown to exhibit anti-tumor activity against multiple cancer models in vitro and in vivo as single agent or in combination with paclitaxel [42, 43]. Although identified as a TTK inhibitor in this study, Hesperadin was previously shown to suppress tumorigenicity through inhibition of Aurora B kinase [44, 45]. Inhibition of TTK via siRNA or CFI-402257, a highly selective TTK inhibitor, promoted apoptosis and inhibited cell proliferation. Such TTK inhibitors are currently being evaluated in clinical trials in TNBC due to their effect on inducing apoptosis and potentiating aneuploidy, possibly through the accelerated progression through mitosis, thus inducing mitotic segregation errors [46]. In addition, CFI-402257-mediated inhibition of TTK in malignant mesothelioma in vitro resulted in overturning the mitotic checkpoint, premature progression through mitosis, marked aneuploidy and mitotic catastrophe [47]. In another study, oral administration of CFI-402257 as single agent or in combination with anti-programmed cell death 1 (PD-1) antibody was proven efficient at tumor inhibition and was well tolerated in mouse models [48]. This small molecule inhibitor is currently in clinical testing as a single agent in advanced solid tumors and in combination with Fulvestrant in advanced breast cancer patients. Additionally, BOS172722, a potent TTK inhibitor, is currently being investigated in a Phase 1/1b study in combination with Paclitaxel in patients with advanced solid malignancies (https://clinicaltrials.gov/). Another small-molecule inhibitor, NMS-P715 has previously been shown to inhibit TTK expression in medulloblastoma, resulting in suppression of cell growth and clonogenic potential, as well as induction of apoptosis, further reaffirming our findings from the current study [49]. There are limited studies on TTKs’ role specifically in CRC, however, one study by Zhang et al., found TTK expression to be higher in CRC patients than in normal tissues, which was related to the unfavorable prognosis of these patients. Their study showed that TTK could activate the PKCα/ERK1/2 signaling pathway to influence proliferation and inactivate the PI3K/AKT pathway to inhibit the expression of MUC2 and TFF3. In basal-like breast cancer, genetic and pharmacological inhibition of TTK was found to radiosensitize cancerous cells through inhibition of homologous recombination [50].

Our gene set enrichment analysis revealed TPX2 and TTK not to work alone, but through a very intricate and complex network involving multiple partners. While currently, there are no available drugs to target TPX2, our gene set enrichment analysis identified numerous players within the TPX2/TTK network. Exploring the druggability of genes from the TPX2/TTK network, we identified several potentially therapeutic targets in CRC, including AURKA, TOP2A, CDK1, ADRM1, TOP1, PSMA7, RRM2, KIF11, CHEK1, CDC7, XPO1, PPAT, PLK4, and BIRC5. In line with those observations, Sillars-Hardebol et al., identified TPX2 and AURKA as two genes located on distinct regions of chromosome 20q that promote 20q amplicon-driven progression of colorectal adenoma to carcinoma [51], concordant with our network analysis. Several of the identified drugs in this study are currently in clinical testing for different cancer types [52]. Wu et al., reported targeting of the AURKA-CDC25C axis to induce synthetic lethality in ARID1A-deficient CRC cells in vitro and in vivo [53]. Numerous other studies explored the therapeutic benefits of topoisomerase inhibitors [54, 55]. While currently there are no such clinical trials for CRC, our data suggests targeting TOP2A, as well as the other identified druggable targets, as a potential therapeutic strategy for CRC.

## Conclusions

Our data, in line with published literature, revealed TPX2 and TTK as CRC essential genes and revealed a complex network involving both genes in patient-derived tumors. Therapeutic targeting of TPX2 and TTK impaired CRC proliferation, colony formation, and growth under 3D conditions. Employing multiple approaches and through integration with patients’ derived transcriptomic data, we identified numerous actionable gene targets based on the TPX2/TTK network, and suggested their potential use in the clinical management of CRC, which warrants further investigations under preclinical and clinical settings.

### Supplementary Information

**Additional file 1: Table S1.** The source of 3D structures of TTK and three interacting drugs.

**Additional file 2: Table S2.** Percentage of cells at each stage of the cell cycle in cells depleted of TPX2, TTK, DDX39A and LRP8.

**Additional file 3: Table S3.** Differentially expressed gene in HT-29 transfected with siTPX2 (2.0 FC, FDR < 0.05).

**Additional file 4: Table S4.** Differentially expressed gene in HT-29 transfected with siTTK (2.0 FC, FDR < 0.05).

**Additional file 5: Table S5.** Differentially expressed gene in HCT116 transfected with siTPX2 (2.0 FC, FDR < 0.05).

**Additional file 6: Table S6.** Differentially expressed gene in HCT116 transfected with siTTK (2.0 FC, FDR < 0.05).

**Additional file 7: Table S7.** Canonical pathway enrichment in differentially expressed genes in TPX2 high vs TPX2 low COAD. Analysis was conducted using IPA.

**Additional file 8: Table S8.** Canonical pathway enrichment in differentially expressed genes in TTK high vs TTK low COAD. Analysis was conducted using IPA.

**Additional file 9: Table S9.** List of enriched genes in TPX2 and TTK network.

**Additional file 10: Table S10.** Gene-Drug interactions for 198 essential genes identified through integration of TPX2/TTK network and CRISPR-Cas9 functional screen data from CRC cell models.

**Additional file 11: Figure S1.** Stage plot. Stage plot correlating the expression of the indicated genes as function of COAD disease stage (I, II, III, and IV). F and p values are indicated on each plot.

**Additional file 12: Figure S2.** Overall Survival (OS) analysis. The eight genes identified in current study were subjected to Kaplan-Myer survival analysis in a cohort of 270 patients with COAD from TCGA. Patients were divided according to median gene expression. The log-rank test was used for curve comparison.

**Additional file 13: Figure S3.** Disease-free Survival (DFS) analysis. The eight genes identified in current study were subjected to Kaplan-Myer survival analysis in a cohort of 270 patients with COAD from TCGA. Patients were divided according to median gene expression. The log-rank test was used for curve comparison.

**Additional file 14: Figure S4.** Effect of targeted depletion of UBE2C, CDCA7, MELK, and NFE2L3 on CFU potential of CRC cells.

**Additional file 15: Figure S5.** Structural modeling and molecular docking. Illustration of the interaction between TTK and BAY-1161909 (a), BAY-1217389 (b), and Hesperadin (c). Pymol was employed to visualize the docking results.

## Data Availability

All data are available in supplementary figures. Additional data can be provided upon request from the corresponding author.

## Abbreviations

CRC: Colorectal cancer
EGFR: Epidermal growth factor receptor
OS: Overall survival
NT: Normal tissues
TPX2: Targeting protein for Xklp2
TTK: TTK Protein Kinase
TPM: Transcript per million
FC: Fold-change
FDR: False discovery rate
DEGs: Differentially expressed genes
IPA: Ingenuity Pathway Analysis
DMEM: Dulbecco’s modified Eagle’s medium
FBS: Fetal bovine serum
CFU: Colony formation unit
PI: Propidium iodide
AO: Acridine orange
EtBr: Ethidium bromide
COAD: Colon adenocarcinoma
GO: Gene ontology
PPI: Protein–protein interaction
TNBC: Triple negative breast cancer
Bay-11: BAY-1161909
Bay-12: BAY-1217389
HES: Hesperadin
