# Supplementary material for: Therapeutic targeting of the TPX2/TTK network in colorectal cancer
Source: Cell Commun Signal. 2023 Sep 28;21:265. doi: 10.1186/s12964-023-01290-2 (PMC10536736; doi:10.1186/s12964-023-01290-2)
Supplement: Supplementary file 2 — Additional file 1: Table S1. The source of 3D structures of TTK and three interacting drugs. [file 12964_2023_1290_MOESM1_ESM.docx]

| **Table S1. The source of 3D structures of TTK and three interacting drugs** | | | |
| --- | --- | --- | --- |
| Synonyms | Name | Source | Link |
| Dual specificity protein kinase TTK | TTK | Alphafold | https://alphafold.ebi.ac.uk/entry/P33981 |
| Empesertib | BAY-1161909 | PubChem | https://pubchem.ncbi.nlm.nih.gov/compound/71599640 |
| Benzamide, N-cyclopropyl-4-[6-(2,3-difluoro-4-methoxyphenoxy)-8-[(3,3,3-trifluoropropyl)amino]imidazo[1,2-b]pyridazin-3-yl]-2-methyl- | BAY-1217389 | PubChem | https://pubchem.ncbi.nlm.nih.gov/compound/78320750 |
| Hesperadine | Hesperadin | PubChem | https://pubchem.ncbi.nlm.nih.gov/compound/135421442 |
