# Supplementary material for: Therapeutic targeting of the TPX2/TTK network in colorectal cancer
Source: Cell Commun Signal. 2023 Sep 28;21:265. doi: 10.1186/s12964-023-01290-2 (PMC10536736; doi:10.1186/s12964-023-01290-2)
Supplement: Supplementary file 3 — Additional file 2: Table S2. Percentage of cells at each stage of the cell cycle in cells depleted of TPX2, TTK, DDX39A and LRP8. [file 12964_2023_1290_MOESM2_ESM.docx]

**Table S2. Percentage of cells at each stage of the cell cycle in cells depleted of TPX2, TTK, DDX39A and LRP8.**

|  | **Sub-G1 phase** | **G1 phase** | **S phase** | **G2 phase** | **Polyploidy**  **(S and G3)** | **Total %** |
| --- | --- | --- | --- | --- | --- | --- |
| HCT116 siControl | **17** | **60** | **8** | **15** | **0** | **100** |
| HCT116 siTPX2 | **55.4** | **14** | **3.6** | **20** | **7** | **100** |
| HCT116 siTTK | **36** | **33** | **7** | **19** | **5** | **100** |
| HCT116 siDDX29A | **26** | **50** | **9** | **15** | **0** | **100** |
| HCT116 siLRP8 | **19** | **57** | **9.5** | **14.5** | **0** | **100** |
| HT-29 siControl | **11** | **64** | **10** | **15** | **0** | **100** |
| HT-29 siTPX2 | **35** | **31** | **7** | **19** | **7** | **100** |
| HT-29 siTTK | **17** | **54** | **9** | **20** | **0** | **100** |
| HT-29 siDDX29A | **18** | **53** | **9** | **20** | **0** | **100** |
| HT-29 siLRP8 | **11** | **58** | **11** | **20** | **0** | **100** |
