# Supplementary material for: Therapeutic targeting of the TPX2/TTK network in colorectal cancer
Source: Cell Commun Signal. 2023 Sep 28;21:265. doi: 10.1186/s12964-023-01290-2 (PMC10536736; doi:10.1186/s12964-023-01290-2)
Supplement: Supplementary file 12 — Additional file 11: Figure S1. Stage plot. Stage plot correlating the expression of the indicated genes as function of COAD disease stage (I, II, III, and IV). F and p values are indicated on each plot. [file 12964_2023_1290_MOESM11_ESM.pdf]

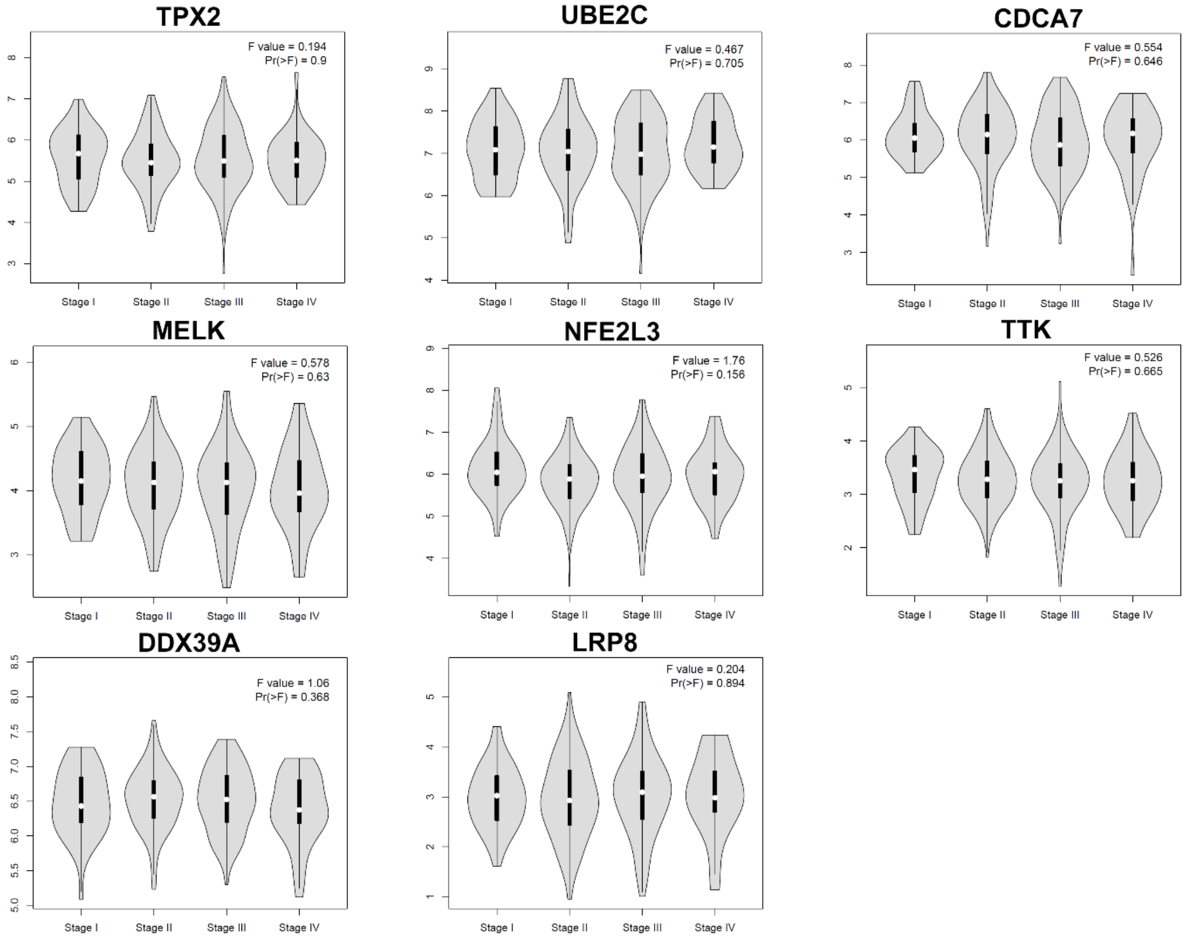


**Figure S1. Stage plot.** Stage plot correlating the expression of the indicated genes as function of COAD disease stage (I, II, III, and IV). F and p values are indicated on each plot.
