# Supplementary material for: Therapeutic targeting of the TPX2/TTK network in colorectal cancer
Source: Cell Commun Signal. 2023 Sep 28;21:265. doi: 10.1186/s12964-023-01290-2 (PMC10536736; doi:10.1186/s12964-023-01290-2)
Supplement: Supplementary file 14 — Additional file 13: Figure S3. Disease-free Survival (DFS) analysis. The eight genes identified in current study were subjected to Kaplan-Myer survival analysis in a cohort of 270 patients with COAD from TCGA. Patients were divided according to median gene expression. The log-rank test was used for curve comparison. [file 12964_2023_1290_MOESM13_ESM.pdf]

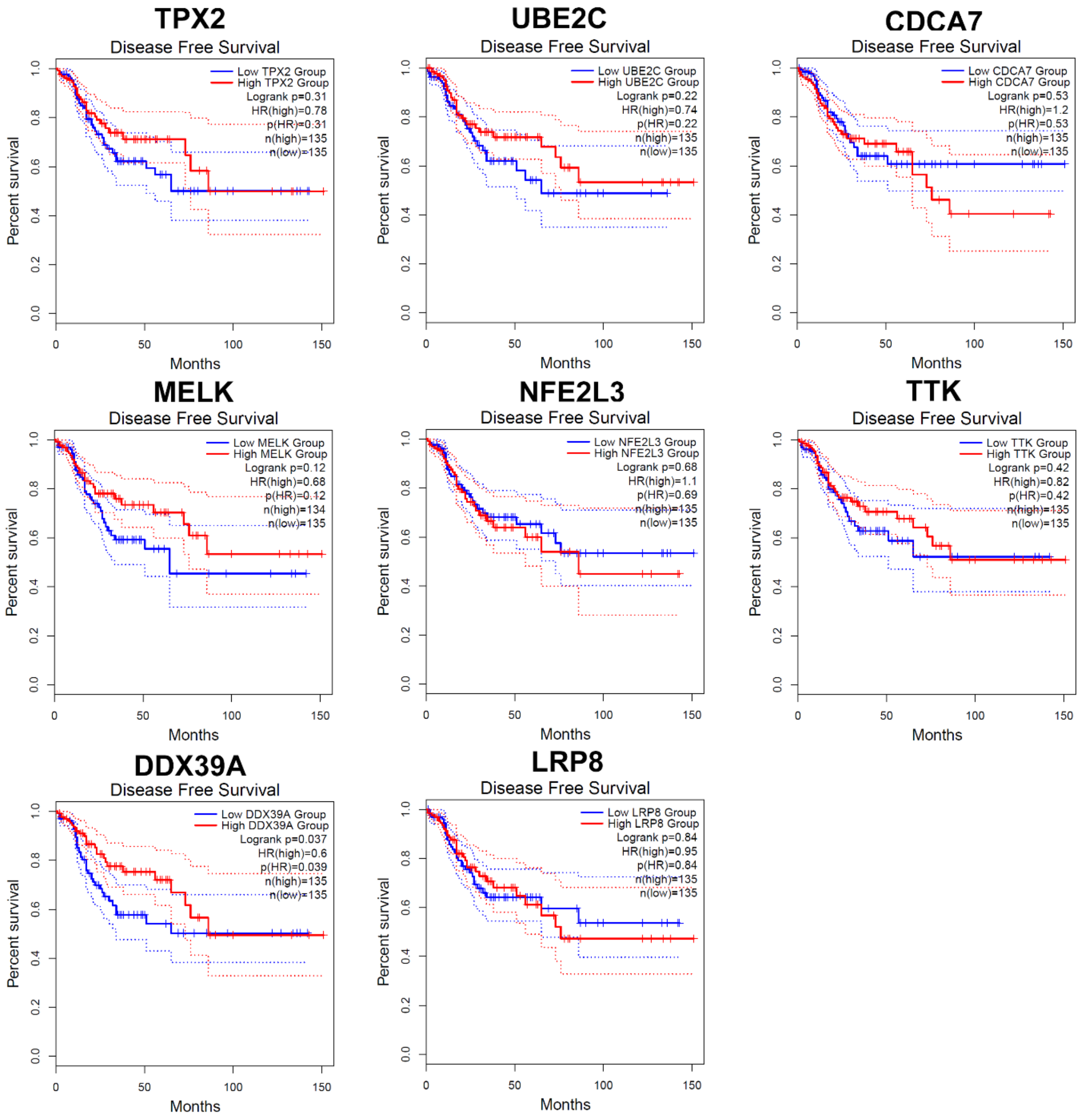

Figure S3. Disease-free Survival (DFS) analysis. The eight genes identified in current study were subjected to Kaplan-Myer survival analysis in a cohort of 270 patients with COAD from TCGA. Patients were divided according to median gene expression. The log-rank test was used for curve comparison.
