# Supplementary material for: Therapeutic targeting of the TPX2/TTK network in colorectal cancer
Source: Cell Commun Signal. 2023 Sep 28;21:265. doi: 10.1186/s12964-023-01290-2 (PMC10536736; doi:10.1186/s12964-023-01290-2)
Supplement: Supplementary file 15 — Additional file 14: Figure S4. Effect of targeted depletion of UBE2C, CDCA7, MELK, and NFE2L3 on CFU potential of CRC cells. [file 12964_2023_1290_MOESM14_ESM.pdf]

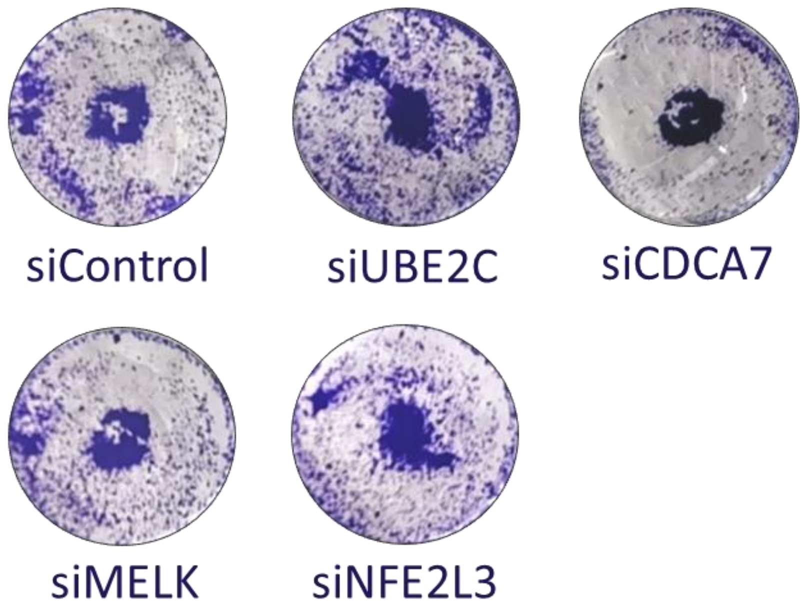

Figure S4. Effect of targeted depletion of UBE2C, CDCA7, MELK, and NFE2L3 on CFU potential of CRC cells. Representative image of HCT116 CRC cells transfected with the indicated siRNAs, then CFU was assessed on day 7 post transfection.
