# Supplementary material for: Therapeutic targeting of the TPX2/TTK network in colorectal cancer
Source: Cell Commun Signal. 2023 Sep 28;21:265. doi: 10.1186/s12964-023-01290-2 (PMC10536736; doi:10.1186/s12964-023-01290-2)
Supplement: Supplementary file 16 — Additional file 15: Figure S5. Structural modeling and molecular docking. Illustration of the interaction between TTK and BAY-1161909 (a), BAY-1217389 (b), and Hesperadin (c). Pymol was employed to visualize the docking results. [file 12964_2023_1290_MOESM15_ESM.pdf]

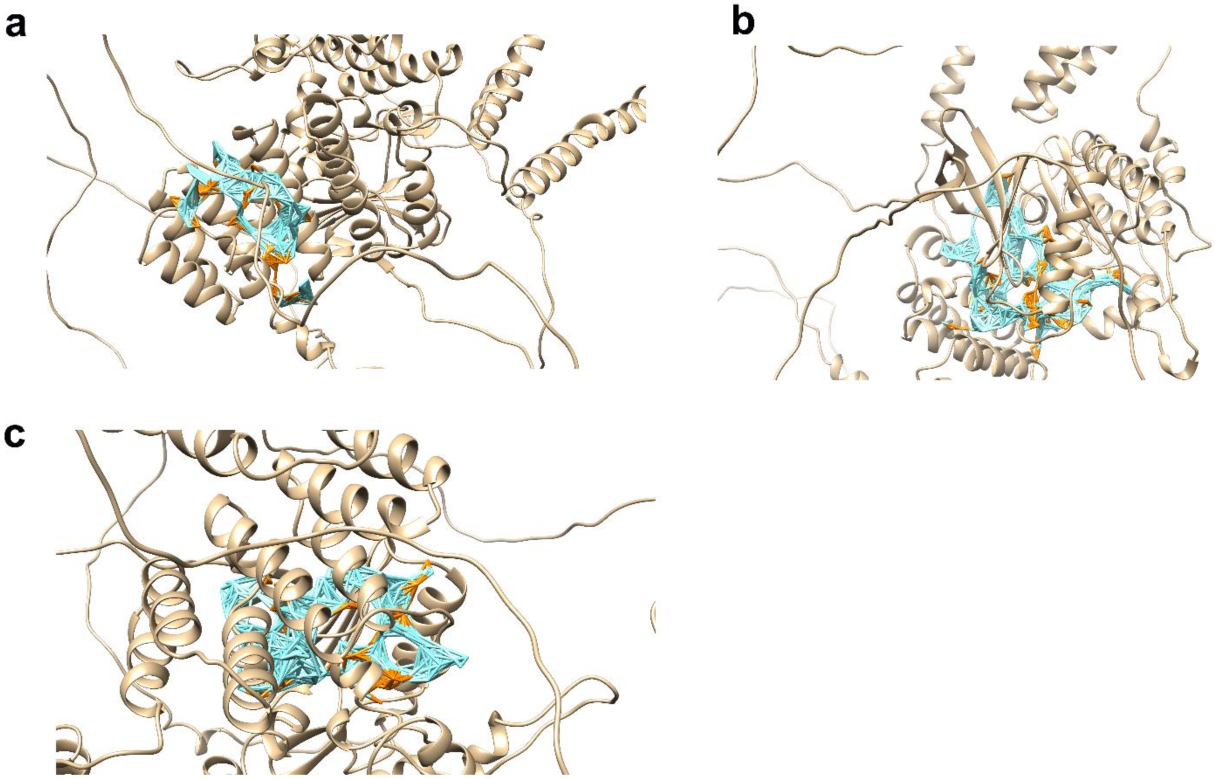

Figure S5. Structural modeling and molecular docking. Illustration of the interaction between TTK and BAY-1161909 **(a)**, BAY-1217389 **(b)**, and Hesperadin **(c)**. Pymol was employed to visualize the docking results.
